# Neonatal Abstinence Syndrome in Infants with Prenatal Exposure to Methadone versus Buprenorphine

**DOI:** 10.3390/children10061030

**Published:** 2023-06-08

**Authors:** Alla Kushnir, Ravi Bhavsar, Emad Hanna, Thomas Hegyi

**Affiliations:** 1 Division of Neonatology, Department of Pediatric, Cooper University Hospital, Camden, NJ 08103, USA; kushir-alla@cooperhealth.edu (A.K.);; 2 Division of Neonatology, Department of Pediatrics, Robert Wood Johnson Medical School, New Brunswick, NJ 08901, USA

**Keywords:** neonatal abstinence syndrome (NAS), opioids, methadone, buprenorphine, outcomes, length of treatment

## Abstract

Neonatal abstinence syndrome (NAS) has been of increasing concern. Studies suggest that prenatal exposure to buprenorphine may be preferred to methadone in regard to neonatal withdrawal. Our aim was to determine whether the incidence and severity of NAS are different between babies prenatally exposed to methadone or buprenorphine in pregnancy. This retrospective analysis of infants ≥ 35-weeks-old exposed to methadone/buprenorphine alone or in conjunction with other substances in utero. They were divided into four groups: 1—methadone alone (Met), 2—buprenorphine alone (Bup), 3 and 4—those exposed to methadone and buprenorphine, respectively, in conjunction with other drugs (Met+ and Bup+). The frequency of NAS treatment, duration of treatment (LOT) and length of stay (LOS) were compared between groups. Of the 290 mothers, 59% were in the Met group, 18% in the Bup group, 14% in the Met or Bup and another opiate group, and 9% took methadone or buprenorphine plus various other substances. Infants born to Met/Met+ mothers had a four-times higher likelihood of developing NAS (*p* < 0.001). There was no difference in the LOS (*p* = 0.08) or LOT (*p* = 0.11) between groups. The buprenorphine treatment in pregnancy decreased the risk of babies developing NAS. However, once the NAS required pharmacological treatment, the type of maternal prenatal exposure did not affect the LOS or LOT.

## 1. Introduction

The prevalence of opioid use disorder (OUD) has increased substantially over the last two decades [1,2]. The rate of NOWS increased nationally from 4.0 to 7.3 per 1000 birth hospitalizations from 2010 to 2017, accompanied by an increase in the rate of opioid-related conditions (e.g., OUD) in mothers at the time of delivery [3]. During the period 1999–2014, the national prevalence of opioid use disorder increased 333%, from 1.5 to 6.5 cases per 1000 delivery hospitalizations [4]. Opioid use in expectant women had close to a five-fold increase from 2000 to 2009 [4,5]. There are a number of perinatal and postnatal factors potentially associated with maternal OUD, such as underuse of preventive care, which may contribute to various adverse infant outcomes [6]. Over the last two decades, the incidence of neonatal abstinence syndrome (NAS) in the U.S. has sharply increased from 1.19 per 1000 hospital births in 2000 to 5.63 in 2012, and there was a five-fold increase in cases from 2004 to 2014 [7]. During the same period, the number of infants treated for NAS in neonatal intensive care units in the U.S. increased five-fold [5,8]. The rise in NAS incidence was primarily due to the increased opioid dependence seen in pregnancy in recent years. By 2009, one NAS infant was born per hour, with an estimated $720 million in hospital charges [5].

Symptoms of NAS occur as a result of the sudden cessation of drugs that the fetus was exposed to in utero [9]. NAS typically presents 48–72 h post-birth and manifests as nervous system irritability, autonomic system dysfunction and gastrointestinal and respiratory abnormalities. Withdrawal signs may begin within 24 h of birth in infants exposed to drugs with a shorter half-life (such as heroin), while withdrawal from methadone and buprenorphine (longer half-life) usually begins between 24 h and 5 days after birth, but can begin as late as at 14 days of age.

Prenatal exposure to opioids is usually associated with multiple risk factors contributing to unfavorable maternal and neonatal outcomes, including a decrease in birth weight and head circumference associated with heroin use during pregnancy [10]. Opioid dependence is not only related to poor medical sequelae, but is also responsible for a substantial and growing portion of resources dedicated to neonates in NICUs and hospitals nationwide [7,8,11,12].

In 1964, methadone, a mu-opioid agonist, was introduced as a replacement treatment for opioid addiction. The use of methadone in subjects who were not pregnant with opioid dependence improved patient outcomes, and its use as part of pharmacotherapy in pregnant women is believed to improve maternal and neonatal prognosis, compared to subjects receiving no treatment at all [13,14,15]. These improvements included higher birth weights, longer gestational ages, and fewer complications compared to infants whose mothers used opioids during pregnancy [10]. In addition, methadone use in the latter part of pregnancy has been shown to affect fetal neurobehavioral outcomes, with split dosing lowering but not improving the effects when compared to single dosing [16,17]. Initially, methadone use during pregnancy was considered unrelated to withdrawal in neonates; however, subsequent experience contradicted this initial assumption. It has now been well-established that exposure to methadone in utero may result in neonatal opioid withdrawal syndrome (NOWS) [3,13,14,18,19,20,21].

Buprenorphine is a partial mu-opioid agonist and kappa-opioid antagonist that has been used to treat opioid dependence in both Europe (1996) and the United States (2002) [22]. Its low intrinsic receptor efficacy due to the partial agonist activity causes its less-than-maximal opioid effect and a reduced risk of overdose compared to methadone [23,24]. The use of buprenorphine during pregnancy is also associated with NOWS. Some studies suggest that it may result in milder manifestations than those seen with methadone [18,25,26,27].

Both buprenorphine and methadone had similar retention in the treatment and suppression of illicit opioid use effects when given at medium or high doses [24]. There were differences seen in neonatal outcomes in the studies comparing the use of methadone and buprenorphine for the treatment of opioid dependency in pregnancy [7,28,29,30]. Prenatal exposure to buprenorphine may be superior to methadone in terms of the infant’s gestational age at birth, birth weight, total morphine dose needed for treatment of symptoms of NAS, duration of NAS treatment and neonatal hospital length of stay [31,32,33,34].

There are emerging data on the differences in the severity of NAS based on the in-utero exposure to methadone vs. buprenorphine [18,22,31,35,36,37,38,39]. The treatment of pregnant women with both of these drugs may lead to neonatal opioid withdrawal syndrome (NOWS) in the neonate. The severity of having withdrawal and developing NAS based on the type of opioid exposure remains unclear.

If there is a concern for, or evidence of, opioid exposure then in utero patients are monitored after birth, and, if signs of withdrawal are noted, a scoring system is used to document the signs and symptoms. A number of scoring systems are available, with the Finnegan Scoring System (FSS) being the most comprehensive one, and the one frequently used to assess NOWS/NAS. The FSS or Modified Finnegan Scoring System (MFSS) is designed to quantify the extent of the withdrawal symptoms in term newborns [40,41,42]. FSS quantifies the signs and symptoms observed in NOWS/NAS. FSS has been validated and is used to monitor the signs of withdrawal as a guide for initiating, adjusting, monitoring and terminating the pharmacological treatment of NAS in newborn infants [43]. The individual symptoms are weighted depending on the symptom. Based on the findings of Zimmermann-Baer et al., scores of 8 or more represent worse withdrawal symptoms and may indicate that pharmacological therapy needs to be used as a treatment of NOWS/NAS [44]. The FSS has been recognized as a reliable scoring tool [45,46,47]. Over the last 5–10 years, the Eat Sleep Console (ESC) has gained much wider acceptance [48,49,50] and is one of the various other scoring systems for NOWS that have been used. Other scoring systems include the Lipsitz Neonatal Drug-Withdrawal Scoring System [51], the Neonatal Withdrawal Inventory [47] and the Neonatal Narcotic Withdrawal Index [43].

The head circumference is one of the predictors of brain volume in children. [28] Prior studies have observed a significant reduction in head growth in utero in chronically opioid-exposed fetuses, resulting in a smaller head circumference at birth [52,53].

We conducted a study to determine whether the incidence of withdrawal from opioids in utero and the severity of NAS are different between babies prenatally exposed to methadone versus buprenorphine, and whether the difference of exposure affects the head circumference.

## 2. Materials and Methods

### 2.1. Study Methods

This was a retrospective review of charts of infants admitted to the Neonatal Intensive Care Unit (NICU) or transitional nursery in an inner city, tertiary level hospital in New Jersey from 2010 to 2017. Inclusion criteria were infants of ≥35 weeks gestational age (GA) whose mothers used prescribed or illicit substances, were on methadone or buprenorphine while pregnant, or had a positive urine drug screen at any point during the current pregnancy. Substances that were disclosed by the mother, or that were documented during maternal or neonatal toxicology screening were recorded.

Pregnant women were divided into 4 groups: 1—treated with methadone alone (Met); 2—treated with buprenorphine alone (Bup); 3—received methadone while still using other drugs (other opiates such as heroin, oxycodone, etc. or any other type of drug) (Met+); and 4—those who received buprenorphine in conjunction with other drugs (other opiates such as heroin, oxycodone, etc. or any other type of drug) (Bup+). Neonates born to mothers who used other opiates alone, or were polysubstance users in combination with other opiates but were not receiving methadone or buprenorphine, were excluded. Neonates who had severe respiratory distress requiring support or had other neurological conditions that made diagnosis of NAS or evaluation with MFSS difficult were excluded. Frequency of documentation of NAS, duration of medical (opiate) treatment for NAS (length of treatment-LOT), and length of stay (LOS) were compared between the 4 groups. NAS was defined as requiring pharmacological treatment for symptoms of withdrawal from opiates based on 3 consecutive Finnegan scores of 8 or greater.

All charts of pregnant women with documented ICD10 code for drug use, abuse, drug exposure, methadone, or buprenorphine treatment (F13–19.10–23, O99.320–324, O35.5XX0, F11.10, F11.20, F11.90, F19.929) were evaluated. The proportion of neonates who required close observation for withdrawal/NAS were documented. Any infants born to mothers identified using the above criteria and admitted to the neonatal intensive care unit (NICU) and/or transitional/intermediate (Trans) unit who had been started on medication to control withdrawal symptoms of NAS were identified from a neonatal database (Neonatal Information System) or electronic medical record (EPIC). All information was documented on a data extraction form. Two members of the team performed all the data collection, and the data were verified by 2 other members of the team, and checked for accuracy. Descriptive data regarding delivery, maternal and neonatal demographics, neonatal outcomes and need for treatment were collected.

Head circumference of the neonates at birth and at discharge was compared between the groups. Head circumference was recorded by the nurse in centimeters on admission and on the day of discharge. Rate of change from admission to discharge was calculated to evaluate head growth. The need for medication treatment for NAS, when it was initiated, what medication was used, and the maximum dosage of that medication were documented and compared between groups. We also recorded LOT and LOS, as well as other short-term neonatal outcomes.

The number of infants diagnosed with NAS who had been started on medication to control withdrawal symptoms was identified as described above. Proportion of mothers treated with methadone versus buprenorphine (±other substances) who had neonates that required treatment was compared between the 4 groups. The severity and duration of NAS symptoms were evaluated by examining the maximum dosage of medication needed and duration of treatment. Head circumference and the percentile of head circumference at birth and at discharge were compared between groups as one of the short-term neonatal outcomes. Secondary factors were also compared between groups. Some comparisons were performed for the 2 groups exposed to buprenorphine vs. methadone, to have a more robust statistical analysis. Statistics were performed using Minitab (Minitab version 17.0, State College, PA, USA). Comparisons between the groups were made using Student’s *t*-tests and Mann–Whitney U tests for continuous data, and chi-squared or Fisher’s exact tests for categorical data. The difference was considered significant at *p* < 0.05.

### 2.2. NAS Treatment 

During a part of the period of data collection there was no standardized protocol in place for the treatment of NAS at our institution. Mothers were encouraged to breastfeed in the absence of illicit substances noted in maternal urine drug screen prior to delivery, as part of the non-pharmacological treatment. All neonates received standard non-pharmacological treatment (such as swaddling, rocking, low-stimulation environment, etc.) while being evaluated for signs of withdrawal and NAS. The general practice for most practitioners was to initiate medication when there were 3 consecutive Finnegan scores of 8 or greater. Finnegan scoring was performed every 3–4 h after feeds (interval depended on the frequency of eating for each baby), and this changed to every 2 h in those who had a Finnegan score greater than 8. Scoring would return to every 3–4 h once 3 consecutive scores below 8 were documented. After the protocol was initiated in 2013, it followed the description noted above.

Morphine would be given orally (PO) when possible; however, in neonates with NAS who were unable to take morphine PO it was given NG/OG. Medical treatment in the form of an opioid (tincture of opium early in the study and morphine sulfate for patients treated after 2013) was started at approximately 0.02–0.04 mg/kg/dose every 3–4 h and increased as needed. Once the protocol was initiated, starting dose was 0.04 mg/kg/dose every 4 h of morphine sulfate. Total opioid dosing was calculated due to multiple types of opioid medication being prescribed during the time of the study.

In the presence of 3 consecutive Finnegan scores greater than 8 or 2 successive scores greater than 13, morphine was increased by 25% of the current dose. When all scores were less than 8 for 24–48 h, morphine was weaned by 10% of the highest amount. Opiate treatment was stopped when the dose was below 0.02 mg/kg/dose every 3 h (or 0.027 mg/kg/dose every 4 h).

In cases where withdrawal symptoms were not well controlled and opioid therapy dose was increased four times or more, phenobarbital was considered and/or initiated as a second-line therapy. Phenobarbital was initiated with a dose of 2.5 mg/kg/dose every 12 h orally. Loading dose was not used when starting phenobarbital; the dose of phenobarbital was typically maintained throughout the stay and for discharge. In patients with more significant withdrawal that was challenging to treat, phenobarbital dose would be weight-adjusted throughout the stay.

Clonidine was not routinely used as a primary or secondary treatment of NAS during the time period examined in this study. Although methadone was already being used by some hospitals to treat NAS, it was not one of the possible medications used to treat NAS at our facility.

## 3. Results

Out of 290 mothers evaluated, 171 (59%) were treated with methadone alone or took methadone with other substances, 49 (17%) were treated with buprenorphine or took buprenorphine with other substances, 44 (15%) took other opioids in addition to methadone or buprenorphine, and 26 (9%) were polysubstance users in addition to methadone or buprenorphine. Of the 171 mothers who received methadone, 96 (56%) took methadone alone (Met). Of the 49 treated with buprenorphine, 44 (90%) took it alone (Bup). All results were verified with a maternal urine drug screen. Out of the 290 babies exposed to methadone or buprenorphine in utero, 207 (71%) developed an NAS that required pharmacological treatment.

See Table 1 for a description of neonates treated for NAS based on the Met, Met+, Bup, and Bup+ groups. In the Met/Met+ group, 81% of neonates required treatment, while in the Bup/Bup+ group, 50% of neonates developed an NAS that required treatment (*p* < 0.001).

Infants born to Met/Met+ mothers had a four-fold higher incidence of NAS (*p* < 0.001) compared to Bup/Bup+. The clinical course of the infants is shown in Table 2. The length of stay (LOS) of babies treated for NAS in the Bup group of mothers was 7 days shorter compared to the Met group (*p* = 0.08). There was no significant difference in the day of life when opiate treatment was started, initial wean was initiated or mean and maximum Finnegan score obtained prior to initiation of treatment (Table 2). The maximum opiate dose per day used for Met/Met+ babies was significantly larger than for the Bup/Bup+ babies. The maximal opiate dose used to treat NAS correlated with the LOS (r = 0.26, *p* < 0.03) and LOT (r = 0.23). Out of the infants treated for NAS, the duration of treatment was not significantly different between groups (*p* = 0.11).

There were significant differences in birth weight between groups (Met 2918 g ± 635, Met+ 2857 g ± 457, Bup 3417 g ± 479 and Bup+ 3099 g ± 449 respectively, *p* <0.001), with neonates in the Bup/Bup+ being significantly heavier than the Met/Met+ ones. There was a significant difference in the type of feeds used (*p* = 0.03). Most neonates received formula, with a minority receiving a combination of formula and breast milk. Only one received breast milk (Table 1) exclusively. There was a significant difference in the rate of exposure to tobacco in utero between the groups, with Met and Met+ having a significantly higher proportion exposed (*p* = 0.04) (Table 1). 

There was no difference between admission head circumference in cm or percentile between groups (*p* = 0.51 and 0.33 respectively). While the head circumference at discharge in cm was not different between groups, there was a significant difference in percentile head circumference at discharge. There were no differences in the rate of head circumference growth (Table 3). 

**Figure 1:**
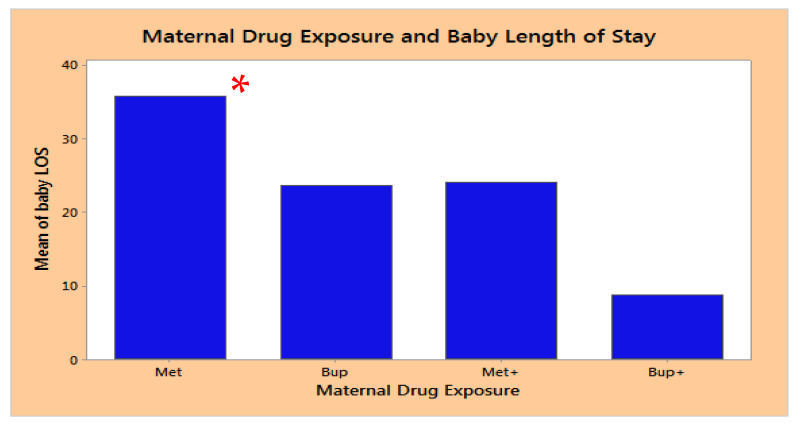
Length of Stay of Neonates with Prenatal Exposure to Methadone vs. Buprenorphine. *—significantly higher LOS in the Met alone group compared to the other groups, *p* < 0.05. Met—methadone exposure alone, Bup—buprenorphine exposure alone, Met+—methadone in conjunction with other drugs or opiates, and Bup+—buprenorphine in conjunction with other drugs.

The mean duration of length of stay (LOS) for all neonates exposed to methadone or buprenorphine in utero, regardless of requiring pharmacological treatment for NAS, was as follows: Met (methadone exposure alone), 35.8 days (95% CI 32.7–38.8); Bup (buprenorphine exposure alone), 23.6 days (95% CI 18.1–29); Met+ (those who took methadone in conjunction with other drugs or opiates), 24 days (95% CI 17.8–30.2); and Bup+ (took buprenorphine in conjunction with other drugs), 8.7 days (95% CI 0.3–17) (See Figure 1). The Met group had a significantly longer LOS compared to the Met+, Bup and Bup+ groups. Those exposed to methadone alone had a significantly longer LOS than those exposed to buprenorphine alone or in combination, or those in the Met+ group (*p* < 0.001) (Figure 1).

**Figure 2:**
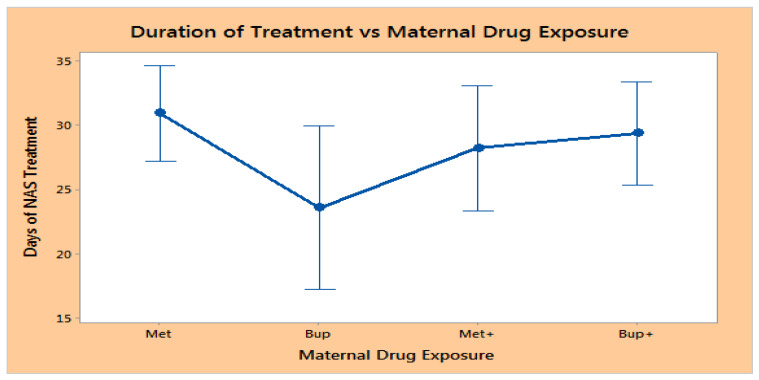
Duration of NAS Treatment Based on Prenatal Exposure. Met—methadone exposure alone, Bup—buprenorphine exposure alone, Met+—methadone in conjunction with other drugs or opiates, and Bup+—buprenorphine in conjunction with other drugs. No significant difference in LOT was noted between groups.

There was not difference in the mean duration of opiate treatment days (with confidence intervals using SD) for neonates who underwent NAS treatment for all neonates exposed to methadone or buprenorphine in utero as seen in Figure 2. The LOT was as follows: Met (methadone exposure alone), 30.9 days (95% CI 27.2–34.6); Bup (buprenorphine exposure alone), 23.6 days (95% CI 17.2–29.9); Met+ (those who took methadone in conjunction with other drugs, opiates or other), 28.2 days (95% CI 23.3–33.4); and Bup+ (took buprenorphine in conjunction with other drugs), 29.3 days (95% CI 25.3–33.4). *p* = 0.11 (Figure 2).

## 4. Discussion

NAS has exhibited a dramatic rise in maternal opioid use disorders in the U.S., with cases more than quadrupling from 1999 to 2014 [51]. This prenatal opioid epidemic is partially due to prescribed opioid medications for pain management or opioid use disorder treatment [54], with the most commonly prescribed opioids being hydrocodone (6.8%), codeine (6.1%) and oxycodone (2.0%) [55]. Opioid dependence is not only associated with a poor medical sequelae, but is also responsible for a substantial and growing portion of resources dedicated to critically ill neonates in NICUs nationwide [7].

Prenatal exposure to opioids is usually associated with unfavorable maternal and neonatal outcomes [56]. In our study, we found that neonates exposed to buprenorphine had a shorter LOT. Similar to our study, prior studies have suggested that buprenorphine-exposed infants have a lower incidence of NAS that requires pharmacological treatment than those exposed to methadone in utero [36]. However, unlike the results found by Kakko, we found that symptoms of withdrawal and severity of withdrawal, as well as the duration of treatment, were similar between groups.

NAS typically presents 2–3 days post-birth [22,57,58] and manifests with tremors, irritability, excessive crying, difficulty sleeping, diarrhea and occasionally seizures [58,59]. Although the effect of exposure to these medications on the neuronal and psychological profile of developing fetuses is unknown, there is a concern that NAS is associated with adverse neurocognitive, behavioral and developmental outcomes [17]. Although parental education may decrease the risk of poor outcomes [17,60], Miller et al. showed that children with a history of NAS had significantly higher rates of language delays at ten years than children without [61].

The clinical characteristics of NAS differ with drug exposure. In methadone-exposed infants, the onset and severity of NAS may vary. Methadone-treated infants may have an earlier start of NAS, even within 24 h of birth, while buprenorphine-exposed infants have shown a delayed onset, sometimes of up to 7 days [62,63].

Initially, infants with NAS are typically managed with non-pharmacological treatment interventions such as breastfeeding, swaddling, rooming-in, environmental control and skin-to-skin contact [64]. In a study by Dryden, breastfed neonates had a shorter length of stay in the hospital than formula-fed neonates or neonates who received formula and breast milk [65]. However, if there is no improvement in symptoms of withdrawal, medications are initiated, with morphine the most commonly used drug for treatment [58]. Successful clinical management is the key to better NAS outcomes [66].

In several investigations, prenatal exposure to methadone compared to buprenorphine resulted in an extended hospitalization, but was often complicated by multiple drug exposure. For example, maternal methadone dose and concomitant in utero exposure to benzodiazepines prolonged the neonatal length of hospital stay [32]. Our results were similar to those of previous authors, where neonates exposed to methadone had a longer length of stay, since they were more likely to require treatment for NAS. However, we did not find significant differences in the duration of treatment of NAS in methadone-exposed versus buprenorphine-exposed infants. The buprenorphine-exposed group required significantly less morphine as seen in other studies [30]. Compared to the length of hospital stay and duration of treatment noted by Metz [34], we found that our population had a more extended hospital stay (35 vs. 25 days) for methadone-exposed infants and (28 vs. 13 days) for buprenorphine-exposed infants. Our length of treatment was approximately 5–6 days shorter than LOS in all groups, bringing the duration of treatment closer to the typical numbers expected. There was no difference in the severity of NAS between the two groups. This was evidenced by the lack of difference in the mean or maximum Finnegan score noted prior to initiation of opioid treatment between groups. This was consistent with following the protocol for all neonates, and initiating medication once the Finnegan scores were higher than 8 three consecutive times, regardless of the day of life of the neonate or neonatal characteristics.

Although there was a significant difference in birth weight between the groups, there was no difference in admission head circumference. Head circumference accurately predicts brain volume in children [28]. Prior studies have observed a significant reduction in head growth in utero for chronically opioid exposed fetuses [50,51]. This impact on brain development is associated with microstructural alteration in major white matter tracts [67]. Buprenorphine-exposed infants presented with similar head circumference, implying a limited protective effect of this partial mu agonist on neuronal compromise, which may occur more frequently with methadone, a full mu agonist [68].

Our findings suggest that neonatal outcomes produced by prenatal exposure to methadone and buprenorphine differ. This difference in the neonatal outcomes may be explained by differences in the placental transfer and the pharmacokinetics of the two medications. Buprenorphine is absorbed more readily from the maternal circulation into placental tissue, but is released less promptly from the placental tissue into the fetal circulation compared to methadone, suggesting that fetal exposure to buprenorphine may be decreased [69].

There was a significant difference in the rate of exposure to tobacco in utero between the groups, with the Met and Met+ groups having a significantly higher proportion exposed. For all groups, it was more common than the incidence seen in pregnant women in the general population in the U.S. (highest prevalence for women aged 20–24 (10.7%), followed by women aged 15–19 (8.5%) and 25–29 (8.2%) [70]. This difference may partially explain the difference in birth weights.

Our retrospective study had several limitations, including uncontrolled confounding variables such as the economic/social status of the pregnant mothers and nutritional status during pregnancy. A further limitation associated with a chart review is the lack of randomization. Due to the pragmatic nature of this study, patients had various in utero exposures, not just to buprenorphine and methadone, but other opiates as well. Based on the variability in exposure, treatment requirements and short-term outcomes may vary between neonates Furthermore, there may have been errors in original data entry, and in the extraction and documentation of data that occurred in the past, which is impossible to verify at the time of the study due to the retrospective nature of the study.

## 5. Conclusions

The incidence of NAS was higher in infants exposed to methadone in utero compared to those exposed to buprenorphine. However, among neonates who required treatment for NAS, there was no significant difference in the severity of NAS, duration of medical treatment or length of stay between the groups. Treating pregnant women with buprenorphine decreased the risk of the baby developing NAS; however, once NAS treatment was initiated, the type of maternal prenatal exposure was not relevant.

## Figures and Tables

**Table 1:** Maternal and infant characteristics of neonates who required treatment for NOWS.

	Methadone Alone (*n* = 54)	Met+ (*n* = 82)	Buprenorphine Alone (*n* = 22)	Bup+ (*n* = 12)	*p*
Birth weight, grams (±SD)	2918 ± 635	2857 ± 457	3417 ± 479	3099 ± 449	<0.001 *
Gestational age, weeks (±SD)	38.9 ± 2.3	38.5 ± 1.8	39.6 ± 1.7	38.7 ± 1.7	0.1
5 min Apgar	9	9	9	9	0.8
Gender					0.2
Male, *n* (%)	34 (63)	38 (48)	13 (59)	8 (67)	
Race, *n* (%)					0.6
Caucasian	47 (87)	72 (88)	20 (91)	11 (92)	
African-American	3 (6)	4 (5)	0	0	
Hispanic	4 (7)	6 (7)	2 (9)	1 (8)	
Delivery					0.3
C/S, *n* (%)	34 (63)	55 (67)	16 (73)	11 (92)	
Food, *n* (%)					0.03 *
BM	1 (2)	0	0	0	
Formula	35 (66)	75 (91)	14 (67)	10 (83)	
Both	17 (32)	7 (9)	7 (33)	2 (17)	
Ethanol, *n* (%)	3 (6)	11(14)	2 (10)	0	0.3
Tobacco, *n* (%)	35 (65)	60 (75)	9 (43)	7 (58)	0.04 *

Met+—neonates exposed to methadone with other substances while in utero, Bup+—neonates exposed to buprenorphine in addition to other substances while in utero; c/s—delivery via cesarian section; food—BM/Formula/Both = breast milk/formula or both used to feed the baby during the stay. Only complete data was used, patients with missing data were not included in the analysis. * = *p* < 0.05.

**Table 2:** Characteristics related to treatment of NOWS.

	Methadone Alone (*n* = 54)	Met+ (*n* = 82)	Buprenorphine Alone (*n* = 22)	Bup+ (*n* = 12)	*p*
Developing NAS, %	85	75	55	15	0.001 *
Max opiate dose, mg/kg/day (SD)	0.2 (0.09)	0.22 (0.12)	0.15 (0.02)	0.16 (0.11)	0.02 *
Mean Finnegan score on initial treatment day (SD)	8.9 (2)	9 (1.8)	9.6 (3.7)	9.1 (3.5)	0.6
Day of life morphine started, day (SD)	3.8 (1.9)	3.3 (1.8)	4 (2)	3.5 (1.6)	0.3
Day of life morphine wean started, days (SD)	13 (7)	12.7 (7.1)	9.8 (7.6)	12.4 (11)	0.4
Maximum Finnegan score prior to morphine start (SD)	11.8 (2.2)	11.7 (2.3)	12 (3.4)	12 (3.1)	1
Mean LOT, days (SD)	29.2 (15)	33 (18)	23.7 (12)	29.3 (19)	0.11
Mean LOS, days (SD)	35.2 (17)	39 (19)	28.3 (12)	34.1 (20)	0.08

Met+—neonates exposed to methadone with other substances while in utero, Bup+—neonates ex-posed to buprenorphine in addition to other substances while in utero; SD—standard deviation; LOT—length of opioid treatment for NAS; LOS—length of stay in the hospital. Comparisons between the groups were made using Mann–Whitney U tests for continuous data and chi-squared or Fisher’s exact tests for categorical data. Only complete data was used, patients with missing data were not included in the analysis. The difference was considered significant at *p* < 0.05 (*).

**Table 3:** Head circumference measurements in neonates treated for NAS.

	Methadone Alone (*n* = 54)	Met+ (*n* = 82)	Buprenorphine Alone (*n* = 22)	Bup+ (*n* = 12)	*p*
Birth HC, cm (SD)	32.7 (2)	32.9 (2)	33.3 (2)	33.3 (2)	0.51
Birth HC% (SD)	24.8 (22)	26.8 (25)	36.4 (31)	27 (30)	0.33
Discharge HC, cm (SD)	35.1 (2)	35.7 (2)	36 (2)	36.1 (2)	0.15
Discharge HC% (SD)	24.4 (19)	29.8 (24.3)	46.8 (27)	39.3 (26)	0.002 *
HC rate of growth, cm/day (SD)	0.08 (0.04)	0.08 (0.04)	0.09 (0.04)	0.09 (0.07)	0.28

HC—head circumference, in centimeters (cm) or percent (%); SD—standard deviation; Birth HC%—percentile measurement of head circumference at birth; * = *p* < 0.05.

## Data Availability

Data are unavailable due to privacy or ethical restrictions.
